# Risk for excessive anticoagulation during hemodialysis is associated with type of vascular access and bedside coagulation testing: Results of a cross-sectional study

**DOI:** 10.3389/fmed.2022.1009748

**Published:** 2022-12-14

**Authors:** Marijke De Troyer, Karl Martin Wissing, Dieter De Clerck, Marie-Laure Cambier, Tom Robberechts, Annelies Tonnelier, Karlien François

**Affiliations:** Division of Nephrology and Hypertension, Universitair Ziekenhuis Brussel (UZ Brussel), Vrije Universiteit Brussel (VUB), Brussels, Belgium

**Keywords:** hemodialysis, heparin, dialyzer, anticoagulation, activated partial thromboplastin time, activated clotting time, extracorporeal circuit clotting, bleeding risk

## Abstract

**Background:**

Recommendations and practice patterns for heparin dosing during hemodialysis show substantial heterogeneity and are scantly supported by evidence. This study assessed the variability in unfractionated heparin (UFH) dosing during hemodialysis and its clinical and biological anticoagulatory effects, and identified explanatory factors of heparin dosing.

**Methods:**

Cross-sectional study assessing UFH dosing, coagulation tests – activated partial thromboplastin time (aPTT) and activated clotting time (ACT) before dialysis start, 1 h after start and at treatment end (4 h) – and measurement of residual blood compartment volume of used dialyzers.

**Results:**

101 patients, 58% male, with a median dialysis vintage of 33 (6–71) months received hemodialysis using a total UFH dose of 9,306 ± 4,079 (range 3,000–23,050) IU/session. Use of a dialysis catheter (*n* = 56, 55%) was associated with a 1.4 times higher UFH dose (*p* < 0.001) irrespective of prior access function. aPTT increased significantly more than ACT both 1 h and 4 h after dialysis start, independent of the dialysis access used. 53% of patients with catheter access and ACT ratio < 1.5, 1 h after dialysis start had simultaneous aPTT ratios > 2.5. Similar findings were present at 1 h for patients with AVF/AVG and at dialysis end for catheter use. No clinically significant clotting of the extracorporeal circuit was noted during the studied sessions. Dialyzer’s blood compartment volume was reduced with a median of 9% (6–20%) without significant effect of UFH dose, aPTT or ACT measurements and vascular access type.

**Conclusion:**

UFH dose adaptations based on ACT measurements frequently result in excessive anticoagulation according to aPTT results. Higher doses of UFH are used in patients with hemodialysis catheters without evidence that this reduces dialyzer clotting.

## Introduction

Hemodialysis is routinely performed using systemic anticoagulation to prevent clotting within the extracorporeal circuit (1, 2). Heparin is the anticoagulant most frequently used because of long standing experience with its use and ease of administration (3). There is no evidence in favor of either unfractionated heparin (UFH) or low-molecular-weight heparin (LMWH) in terms of efficacy and bleeding complications (4, 5). Dose recommendations and practice patterns for heparin administration during hemodialysis show substantial heterogeneity and are scantly supported by scientific research (6). The British recommendations for UFH dosing during hemodialysis do not specify the recommended loading dose (7). European guidelines suggest a loading dose of 50 IU UFH/kg (8). The recommended maintenance dose for UFH use during hemodialysis varies between 500 and 1,500 IU/hour, with the heparin administration to be stopped prior to the end of the session in case of an arteriovenous fistula or graft (AVF/AVG) to prevent prolonged bleeding of the needling site (6–8). US hospitals use a variety of empirical UFH protocols based on local practice (9). Although the rationale for the recommendations is unclear, prescribed doses of UFH during hemodialysis most likely correspond to an increase of activated partial thromboplastin time (aPTT) or whole blood activated clotting time (ACT) of 1.5–2 times baseline value (10). All practice recommendations agree to reduce or avoid systemic heparinization during hemodialysis for patients presenting an increased risk of bleeding (6).

Previous attempts in creating a general framework for UFH dose determination include pharmacodynamic modelling and population-based statistical techniques (3, 11). Although not adopted in the clinical setting thus far, these techniques support the assumption that basing UFH dose on patient weight and session duration alone inevitably leads to excessive or inadequate anticoagulation in some patients (3).

aPTT and ACT assess the intrinsic and common coagulation pathways. aPTT testing requires a laboratory setting, varies according to the reagent used and has a 2-h turnaround time, which makes it unsuited for quick dose adjustments (6, 9). ACT measures anticoagulation effect by bedside point-of-care testing. ACT testing, however, has not been standardized and lacks in accuracy, especially at low heparin levels (6, 12). The drawbacks of biological monitoring of the unfractionated heparin effect have contributed to a pragmatic stance concerning dose adjustments (9). In clinical practice, visual assessment of clotting within dialyzer and venous air detector chamber, excessively raised transmembrane pressure gradients, dialysis adequacy parameters below target or prolonged bleeding time of the arteriovenous access puncture site may indicate insufficient or excessive anticoagulation. These clinical signs then guide the nephrologist or nurse to adjust UFH dose, with or without additional ACT monitoring (6, 13, 14).

This study was conducted to assess the variability in UFH dosing and its clinical and biological anticoagulatory effects during hemodialysis in a prevalent cohort, and to identify explanatory factors of heparin dosing.

## Materials and methods

### Study design and study population

**FIGURE 1:**
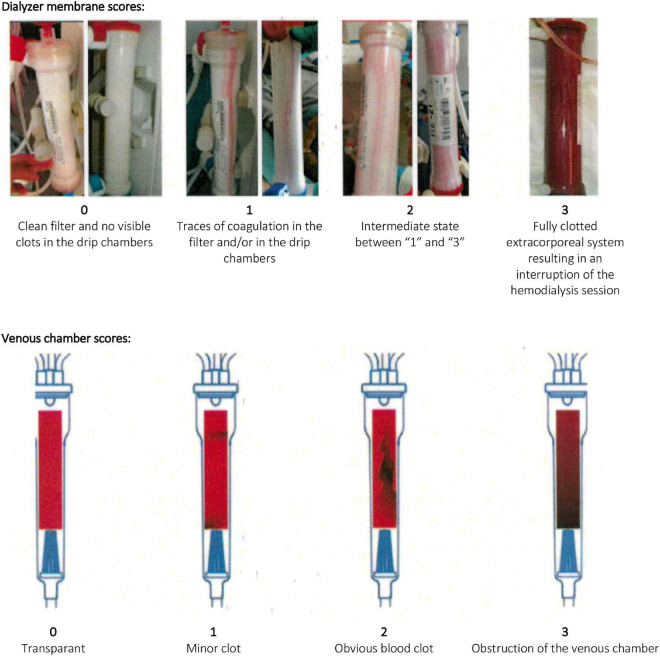
Semiquantitative scoring system for dialyzer membrane and venous chamber. During the quality improvement project, the hemodialysis nurses scored both the dialyzer and the venous chamber after each hemodialysis session according to the above scoring scheme. Scores were noted on the electronic data sheet of the hemodialysis session.

In June 2019, a continuous quality improvement project was set up in the HD department at Universitair Ziekenhuis Brussel given the ascertainment of high heparin doses used during hemodialysis in several patients. Within this project, clotting complications and post puncture bleeding of arteriovenous access needling sites were assessed during every hemodialysis session using standardized semiquantitative scores (Figure 1) and every monthly blood work between June and December 2019 was extended with aPTT before dialysis start, 1 h after dialysis start and at session end. For as many patients as possible, ACT testing was performed simultaneously with aPTT measurements during one hemodialysis session. In addition, each participant had a blood compartment volume measurement of used dialyzers at the end of a dialysis session with monthly blood work.

Our unit’s standard of care of systemic anticoagulation during hemodialysis is the use of UFH. Upon the start of chronic hemodialysis, UFH is administered according to a standing order using a loading dose of 2,500 IU UFH and a maintenance dose of 1,400 IU UFH/hour until completion of the hemodialysis session in case of catheter use and until 1 h before hemodialysis session end in case of arteriovenous (AV)-access. Both loading dose, maintenance dose and infusion time are adjusted in case of thrombotic complications of the extracorporeal circuit, access dysfunction or bleeding complications. ACT is measured during the first hemodialysis sessions for incident hemodialysis patients and in case of thrombotic or bleeding complication in prevalent hemodialysis patients. UFH dose is adjusted whenever ACT is below 180 s or above 250 s. The quality improvement project did not include to adjust UFH dose based on aPTT results.

The dialyzers used during the quality improvement project were Phylter HF SD 1.7, Phylter HF SD 2.2 (both Medtronic, USA), TS Toray 1.8, TS Toray 2.1 (both Toray Industries Inc., Tokyo, Japan) and BG Toray 1.8 (Toray Industries Inc., Tokyo, Japan). Information on the original blood compartment volume of the used dialyzers was retained from the product leaflet.

A study protocol and statistical analysis plan for a cross-sectional study were subsequently designed for a more detailed analysis of the data generated during the quality improvement project. This study was set up to assess UFH dose variability within the prevalent hemodialysis population, to evaluate biological anticoagulant effects of UFH and clinical signs of insufficient or excessive anticoagulation and to identify explanatory factors of UFH dosing. The Ethics Committee of Universitair Ziekenhuis Brussel approved the study (Reference 2019/428) and waived the need for informed consent as study data were retrospectively collected.

This cross-sectional study included patients having participated in the quality improvement project between June and December 2019. For each patient, data were analyzed concerning the hemodialysis sessions with simultaneous ACT and aPTT monitoring or with simultaneous monitoring of aPTT and a blood compartment volume measurement of the used dialyzer. Hemodialysis sessions performed without systemic anticoagulation were excluded from analysis.

### Study endpoints

Data on UFH loading dose, maintenance dose and infusion time during the hemodialysis session were collected. aPTT was measured in the central lab (2.9 ml sodium citrate blood collector) using turbidimetric clot detection on an ACL Top Family machine. ACT assessment was performed bedside using a whole blood sample and an ACTester (Quest Medical, Allen, TX, USA), a photometric ACT test system which uses celite as activator. The increment of aPTT and ACT increase was normalized to baseline value (ratios t1/0 and t4/0) among individual patients to get dimensionless variables both assessing the effect of UFH allowing comparison of both measures. Ratios of aPTT and ACT at 1 and 4 h after dialysis start over baseline were categorized according to their therapeutic level: ratios < 1.5 were considered below therapeutic range (“insufficient”), ratios between ≥ 1.5 and ≤ 2.5 as therapeutic (“adequate”) and ratios > 2.5 being supratherapeutic (“excessive”) (3, 15).

Semiquantitative clotting scores of the dialyzer membrane or the venous chamber reflected (0) clean filter or no visible clots in the drip chamber, (1) traces of coagulation in the filter or in the drip chamber, (2) intermediate state between 1 and 3 or (3) a fully clotted extracorporeal circuit resulting in an interruption of the HD session (Figure 1). Arteriovenous access sites were inspected 15 min after needle removal and puncture sites scored (0) if no bleeding, (1) if limited oozing or (2) if excessive bleeding was present. The residual blood compartment volume of the dialyzer (mL) after the hemodialysis session was measured by a Renatron-II 100 series dialyzer reprocessing system. This volume was expressed as a proportion of the original blood compartment volume of the dialyzer (%) and considered an estimate of the amount of blood compartment loss of the dialyzer due to clotting.

### Statistical analysis

A statistical analysis plan was set up prior to data collection. Descriptive statistics were applied. Two sample *t*-test assessed differences between groups and paired *t*-test evaluated differences within matched samples. Contingency tables and chi-square hypothesis tests assessed differences in proportions between aPTT and ACT ratios and differences in proportions between aPTT ratios and semiquantitative clotting scores. The difference between aPTT and ACT testing was assessed using Bland and Altman analysis. Variables associated with UFH dose or small solute clearance were analyzed in a multivariable linear regression model. The anonymized dataset is publicly available in a data repository (https://doi.org/10.5281/zenodo.5007102).

## Results

### Study population

**FIGURE 2:**
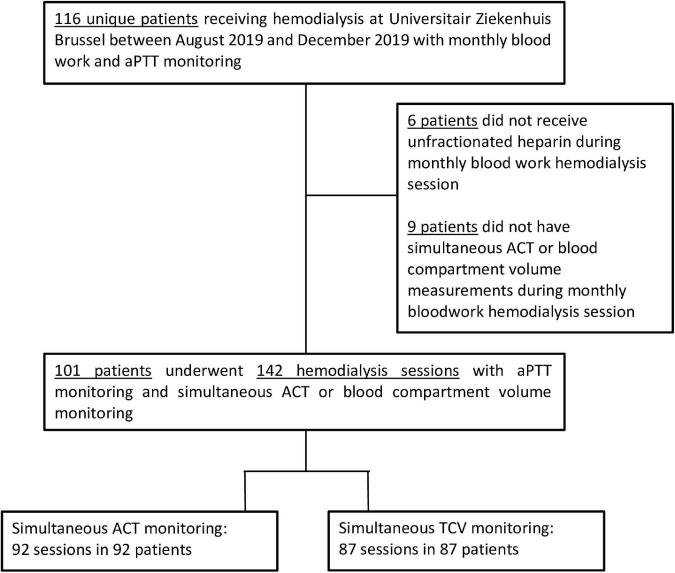
Hemodialysis session selection for study inclusion. This study included 101 patients with hemodialysis session data on 92 sessions with simultaneous activated partial thromboplastin time (aPTT) and activated clotting time (ACT) monitoring and 87 sessions with simultaneous aPTT and blood compartment volume measurements.

Patient and hemodialysis session selection for cross sectional analysis is described in Figure 2. Characteristics of the 101 study subjects are described in Table 1.

**TABLE 1:** Relevant demographic, medical and drug therapy data of the study population.

	*n* = 101
Age (years)	69 ± 13
Gender, male (*n*, %)	59 (58)
Weight (kg)	74 ± 18
**Race (*n*, %)**	
Caucasian	61 (60)
Black African	14 (14)
Northern African	24 (24)
Asian	2 (2)
**ESKD etiology (*n*, %)**	
Diabetes mellitus	17 (17)
Hypertensive nephrosclerosis	22 (22)
Combination of diabetic and vascular kidney failure	23 (23)
Glomerulonephritis	7 (7)
Tubulointerstitial nephropathy	7 (7)
Genetic disease	9 (9)
Other	16 (16)
Dialysis vintage (months)	33 (6–71)
**Vascular access type (*n*, %)**	
AV fistula	41 (41)
AV graft	4 (4)
Catheter	56 (55)
History of HD vascular access dysfunction (*n*, %)	33 (33)
Treated diabetes mellitus (*n*, %)	47 (47)
**Use of antiplatelets (*n*, %)**	
None	36 (36)
Acetylsalicylic acid	52 (51)
Clopidogrel	1 (1)
Combination	12 (12)
**Use of anticoagulants (*n*, %)**	
None	85 (84)
Anti-vitamin K	14 (14)
LMWH	2 (2)

Data are given as mean ± SD, proportions (%) or median (IQR) when appropriate. ESKD, end-stage kidney disease; AV, arteriovenous; HD, hemodialysis; LMWH, low molecular weight heparin.

History of HD vascular access dysfunction was defined as arteriovenous fistula thrombosis ever, catheter replacement for thrombotic dysfunction ever, or urokinase use because of access thrombosis during the last 3 months.

### Variability in UFH dose

**FIGURE 3:**
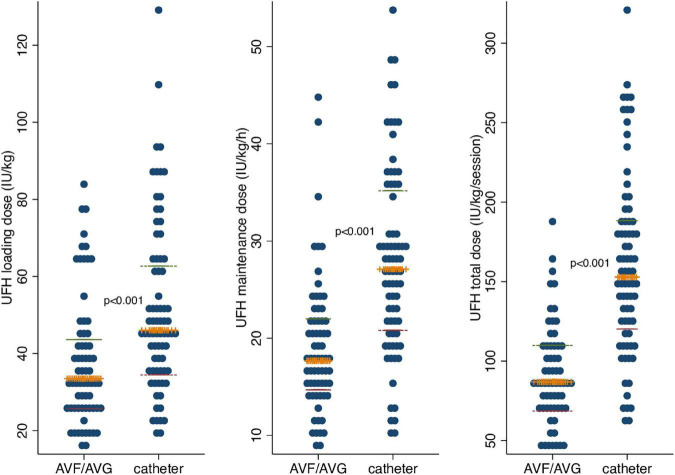
UFH loading, maintenance and total dose within study subjects, according to vascular access. UFH, unfractionated heparin; IU, international units; kg, kilogram; h, hour; AVF/AVG, arteriovenous fistula/arteriovenous graft. The orange “+”-signs represent the median, the green dashed lines represent the 75th percentile and the red dashed lines represent the 25th percentile.

Across individual patients, mean UFH loading dose was 3,317 ± 1,494 IU/session (range 1,250–7,500 IU/session) [45 ± 22 IU/kg/session] and maintenance dose 1,712 ± 649 IU/h (range 700–4,200 IU/h) [24 ± 9 IU/kg/h]. Maintenance dose was administered until completion of the 4-h hemodialysis session in case of catheter use and stopped 75 ± 20 min before dialysis session end for patients with an AV-access. Overall mean total UFH dose was 9,306 ± 4,079 IU/session (range 3,000–23,050 IU/session) [128 ± 58 IU/kg/session]. Patients with a catheter vascular access (*n* = 56, 55%) received significantly higher (×1.4) loading and maintenance UFH doses (*p* 0.001): 52 ± 23 vs 37 ± 17 IU/kg/session loading dose and 27 ± 9 vs 19 ± 7 IU/kg/h maintenance dose in case of catheter or AV-access, respectively (Figure 3). UFH dose did not differ between patients presenting a history of access dysfunction or not, although a trend for higher UFH doses was noted in patients with a history of access dysfunction and dialyzed using a catheter access (*p* = 0.055; Table 2). Multiple linear regression analysis confirmed access type as a significant predictor of UFH dose after adjustment for access dysfunction (*p* < 0.001). UFH dose did not differ according to gender, diabetes status, CRP ≤ or >5 mg/L, serum albumin < or ≥ 35 g/dl, hemoglobin tertiles, antiplatelets or anticoagulants use and prior vascular disease history (Table 2). Access type explained 35% of the variation of total UFH dose (*R*^2^ = 0.35 by one-way analysis of variance).

**TABLE 2:** Total UFH dose per hemodialysis session according to patient characteristics.

		Total UFH dose (IU/kg/session)	*p*-value^1^
Gender	Male	126 ± 52	0.7
	Female	131 ± 67	
Diabetes mellitus	Yes	130 ± 51	0.8
	No	127 ± 64	
CRP	≤5 mg/L	126 ± 58	0.8
	>5 mg/L	129 ± 59	
Albumin	<35 g/dl	136 ± 68	0.5
	≥35 g/dl	126 ± 56	
Hemoglobin	Lowest tertile	125 ± 43	0.96
	Middle tertile	129 ± 62	
	Highest tertile	130 ± 66	
Antiplatelet therapy	Yes	129 ± 59	0.8
	No	126 ± 58	
Anticoagulant therapy	Yes	137 ± 75	0.5
	No	127 ± 55	
**History of vascular access dysfunction**			
AVF/AVG	Yes (*n* = 5)	67 ± 13	0.1
	No (*n* = 38)	92 ± 33	
Catheter	Yes (*n* = 27)	173 ± 55	0.055
	No (*n* = 29)	145 ± 55	

Total UFH dose expressed in IU/kg/session is depicted as mean ± SD.

CRP, c-reactive protein; AVF/AVG, arteriovenous fistula/arteriovenous graft.

^1^Hypothesis testing of the difference between the different categories of the variable using unpaired *t*-test (gender, diabetes mellitus, CRP, serum albumin, antiplatelet therapy, anticoagulant therapy, and history of vascular access dysfunction) or one-way anova (hemoglobin tertiles).

### Biological monitoring of unfractionated heparin dose

**FIGURE 4:**
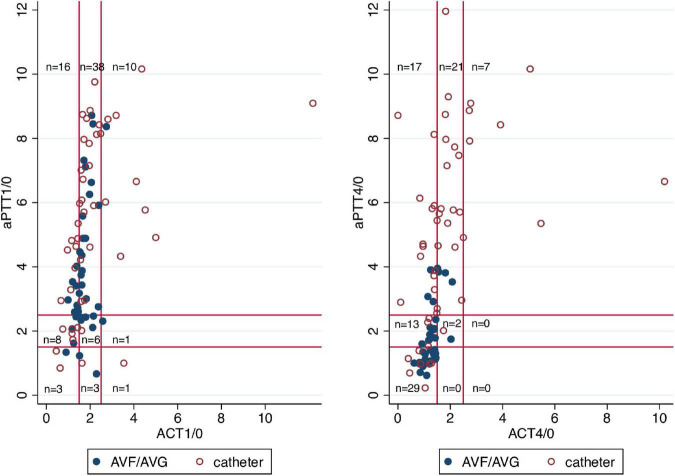
Relation between aPTT and ACT ratios both 1 h (left panel) and 4 h (right panel) after dialysis start. The red horizontal and vertical reference lines represent the ACT and aPTT ratios of × 1.5 and × 2.5, defining the therapeutic range of anticoagulation. The blue dots represent patients with an arteriovenous fistula or graft, the red circles represent patients with a catheter access. The shorter infusion time of unfractionated heparin in case of AVF/AVG use explains the shift toward lower left for AVF/AVG patients in the right graph. aPTT, activated partial thromboplastin time; ACT, activated clotting time; AVF/AVG, arteriovenous fistula/arteriovenous graft.

The aPTT ratio was significantly higher than the ACT ratio both 1 and 4 h after dialysis start, independent of the dialysis access used (Tables 3, 4 and Figure 4).

**TABLE 3:** Activated partial thromboplastin time (aPTT) and activated clotting time (ACT) ratios at t1 and t4 according to dialysis vascular access.

		aPTT ratio	ACT ratio	*p*-value^1^
**t1/0**				
	Catheter	5.3 (4.5–6.1)	2.2 (1.7–2.7)	<0.001
	AVF/AVG	3.9 (3.2–4.6)	1.7 (1.6–1.8)	<0.001
	p-value^2^	0.007	0.1	
**t4/0**				
	Catheter	5.1 (4.3–5.9)	1.9 (1.4–2.3)	<0.001
	AVF/AVG	1.8 (1.5–2.1)	1.3 (1.2–1.4)	<0.001

This table shows the aPTT and ACT ratio results expressed as mean (95%CI) according to the vascular access and according to the time after dialysis start the ratios were calculated.

aPTT, activated partial thromboplastin time; ACT, activated clotting time; AVF/AVG, arteriovenous fistula/arteriovenous graft.

t1/0 and t4/0 refers to the measurements at 1 and 4 h after dialysis start, respectively over baseline value.

^1^Hypothesis testing of the difference between aPTT and ACT ratio using paired *t*-test.

^2^Hypothesis testing of the difference in coagulation test ratio between catheter and AV-access using unpaired *t*-test.

**TABLE 4:** Activated partial thromboplastin time (aPTT) and activated clotting time (ACT) results at baseline, during and after hemodialysis.

	Before dialysis start	1 h after dialysis start	4 h after dialysis start
**AVF/AVG**			
aPTT (s)	31 (29–35)	109 (73–182)	46 (36–61)
ACT (s)	83 (71–95)	140 (122–164)	107 (93–123)
**Catheter**			
aPTT (s)	34 (30–44)	250 (134 = 250)	248 (135 = 250)
ACT (s)	101 (89–133)	183 (133–238)	160 (131–220)

aPTT and ACT results in seconds expressed as median (interquartile range) according to sample time and vascular access.

aPTT, activated partial thromboplastin time (normal range 22.2–34.4 s); ACT, activated clotting time (normal range 70–120 s); AVF/AVG, arteriovenous fistula/arteriovenous graft.

**FIGURE 5:**
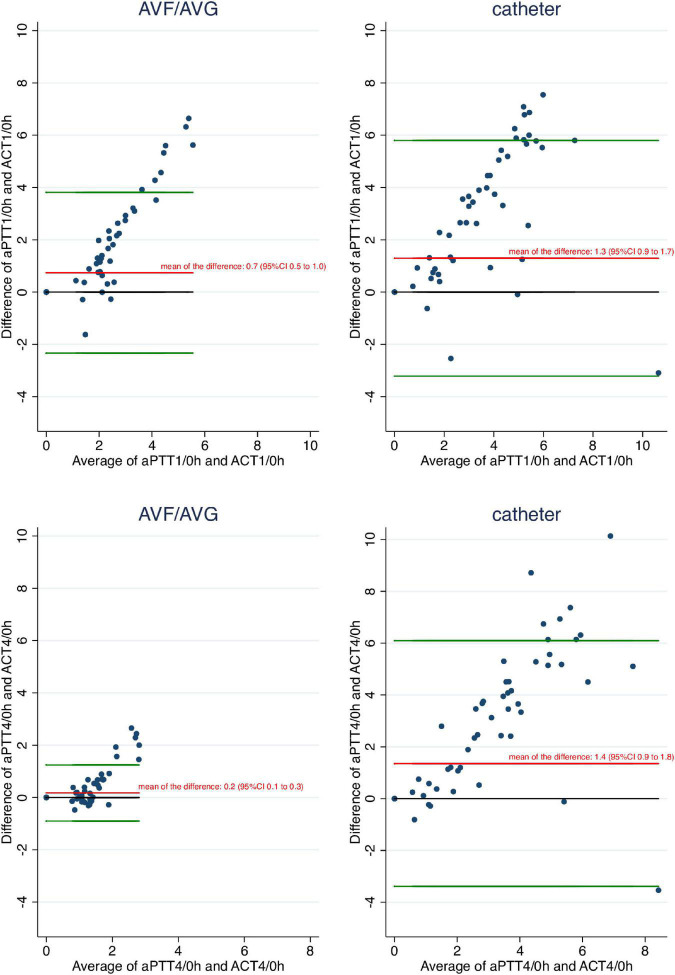
Bland and Altman analysis comparing aPTT and ACT ratios according to vascular access. These Bland and Altman graphs show the difference between aPTT and ACT ratios over the average of aPTT and ACT ratios. Bland and Altman plots are shown for aPTT and ACT values 1 h after dialysis start over baseline (upper panel) and for aPTT and ACT values 4 h after dialysis start over baseline (lower panel) according to access type.

Table 5 shows the proportions of patients presenting adequate, excessive or insufficient anticoagulated state according to the aPTT or ACT ratio and the vascular access used. The lack of agreement between aPTT and ACT is confirmed and quantified by Bland and Altman analysis. The mean of the differences between aPTT and ACT ratios was positive both for 1 and 4 h ratios and irrespective of the vascular access used (Figure 5). When the average of aPTT and ACT gets larger, the difference between aPTT and ACT increases reflecting a larger increase of aPTT relative to ACT with incremental anticoagulation.

**TABLE 5:** Adequate, excessive or insufficient anticoagulated state according to the activated partial thromboplastin time (aPTT) or activated clotting time (ACT) ratio and according to dialysis vascular access.

AVF/AVG		aPTT 1/0			
		<1.5	≥1.5 and ≤ 2.5	>2.5	Total
ACT 1/0	<1.5	1	3	8	**12 (31)**
	≥1.5 and ≤ 2.5	2	5	18	**25 (64)**
	>2.5	0	1	1	**2 (5)**
	Total	**3 (8)**	**9 (23)**	**27 (69)**	**39 (100)**

		**aPTT 4/0**			
		**<1.5**	**≥1.5 and ≤2.5**	**<2.5**	**Total**

ACT 4/0	<1.5	**22**	**10**	**3**	**35 (88)**
	≥1.5 and ≤ 2.5	**0**	**1**	**4**	**5 (13)**
	>2.5	**0**	**0**	**0**	**0 (0)**
	Total	**22 (55)**	**11 (27.5)**	**7 (17.5)**	**40 (100)**

**Catheter**		**aPTT 1/0**			
		**<1.5**	**≥1.5 and ≤2.5**	**>2.5**	**Total**

ACT 1/0	<1.5	2	5	8	**15 (32)**
	≥1.5 and ≤2.5	1	1	20	**22 (47)**
	>2.5	1	0	9	**10 (21)**
	Total	**4 (8)**	**6 (13)**	**37 (79)**	**47 (100)**

		**aPTT 4/0**			
		**<1.5**	**≥1.5 and ≤2.5**	**>2.5**	**Total**

ACT 4/0	<1.5	**7**	**3**	**14**	**24 (49)**
	≥1.5 and ≤2.5	**0**	**1**	**17**	**18 (37)**
	>2.5	**0**	**0**	**7**	**7 (14)**
	Total	**7 (14)**	**4 (8)**	**38 (78)**	**49 (100)**

This cross-table shows the numbers of patients categorized by access type and according to ratios of aPTT and ACT at 1 and 4 h after dialysis start over baseline. ACT and aPTT ratios were categorized according to their therapeutic level: ratios < 1.5 were considered below therapeutic range (“insufficient”), ratios between ≥ 1.5 and ≤ 2.5 as therapeutic (“adequate”) and ratios > 2.5 being supratherapeutic (“excessive”). The shorter infusion time of unfractionated heparin in case of AVF/AVG use explains the shift toward lower aPTT and ACT ratios 4 h after dialysis start.

aPTT, activated partial thromboplastin time; ACT, activated clotting time; AVF/AVG, arteriovenous fistula/arteriovenous graft.

### Clotting of the extracorporeal circuit

The proportions of semiquantitative clotting scores were not associated with UFH dose or aPTT ratios at 4 h (Table 6).

**TABLE 6:** Semiquantitative clotting scores of the extracorporeal circuit and the AV-access site after dialysis.

Clotting score of the dialyzer	*n* (%)	Total UFH dose (IU/kg/sess)	aPTT t4/0
Clean filter	15 (16)	119 ± 40	2.7 ± 1.8
Traces of coagulation in the filter	60 (64)	130 ± 64	3.7 ± 3.0
Intermediate state between previous and next score	19 (20)	126 ± 49	3.6 ± 2.3
Fully clotted ECC with interruption of the HD session	0 (0)	NA	NA

**Clotting score of the venous chamber**	***n* (%)**	**Total UFH dose (IU/kg/sess)**	**aPTT t4/0**

No visible clots in the drip chambers	63 (67)	131 ± 58	3.8 ± 2.8
Traces of coagulation in the drip chambers	27 (29)	123 ± 59	3.2 ± 2.5
Intermediate state between previous and next score	4 (4)	91 ± 29	1.7 ± 1.6
Fully clotted ECC with interruption of the HD session	0 (0)	NA	NA

**Puncture site bleeding score (for AVF/AVG patients only)**	***n* (%)**	**Total UFH dose (IU/kg/sess)**	**aPTT t4/0**

No bleeding 15 min after needle removal	29 (71)	90 ± 31	1.9 ± 1.3
Limited oozing 15 min after needle removal	9 (22)	82 ± 40	1.4 ± 0.5
Excessive bleeding 15 min after needle removal	3 (7)	101 ± 10	1.8 ± 1.0

ECC, extracorporeal circuit; IU, international units; sess, session.

**FIGURE 6:**
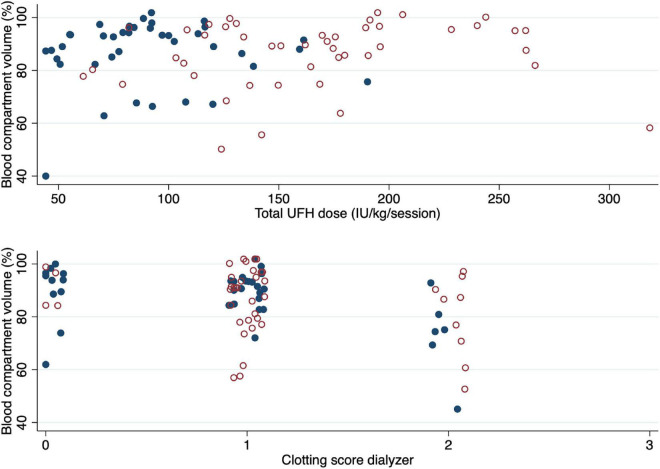
Relation of blood compartment volumes of used dialyzers with UFH dose and dialyzer clotting score. The blood compartment volume of used dialyzers, expressed as the proportion (%) of the measured volume after use over the theoretical volume, is shown according to the total dose of unfractionated heparin administered during the hemodialysis session (upper panel) and the clotting score of the dialyzer (lower panel). The blue dots represent patients with an arteriovenous fistula or graft, the red circles represent patients with a catheter access.

Relative median blood compartment volume of used dialyzers was 91% (IQR 84–94%) and 90% (IQR 79–94%) for AV-access and catheter vascular access, respectively (*p* = 0.8). Access type was not associated with a blood compartment volume loss of 20% or more after dialysis according to multivariable logistic regression analysis. Loss of blood compartment volume of the dialyzer was not associated with total UFH dose (Table 7 and Figure 6). A large variation in blood compartment volume loss was noted for each category of the semiquantitative clotting score of the dialyzer (Figure 6).

**TABLE 7:** Total UFH dose and aPTT4/0 ratios according to blood compartment volume results.

		Blood compartment volume		*p*-value^1^
		<80%	≥80%	
		*n* = 22	*n* = 65	
Total UFH dose	Overall	125 ± 59	133 ± 62	0.6
(IU/kg/session)	AVF/AVG	103 ± 42	86 ± 30	0.2
	catheter	140 ± 66	175 ± 51	0.06
aPTT4/0	Overall	3.1 ± 2.7	3.5 ± 2.7	0.6
	AVF/AVG	1.6 ± 1.3	1.8 ± 1.2	0.7
	catheter	4.2 ± 3.0	5.1 ± 2.8	0.3

Total session dose of unfractionated heparin (UFH) (IU/kg/session) and aPTT4/0 ratio are expressed as mean ± SD according to the loss in blood compartment volume of the dialyzer after use normalized for its original volume.

aPTT, activated partial thromboplastin time; ACT, activated clotting time;

AVF/AVG, arteriovenous fistula/arteriovenous graft.

^1^Hypothesis testing of the difference between categories of blood compartment loss using unpaired *t*-test.

## Discussion

Our study provides insight in UFH dosing in a single-center prevalent hemodialysis population and identifies vascular access type as an explanatory factor for heparin dose variability. To our best knowledge, this is the first study directly comparing the effect of unfractionated heparin administration during hemodialysis on aPTT and ACT results.

Our patients received UFH in a large range of loading and maintenance doses and a quarter of the patients received more than 12,150 IU UFH per hemodialysis session. Remarkably, the maintenance UFH doses were above recommended dose in the majority of patients, irrespective of the vascular access used, while UFH loading doses were in line with ERA-EDTA and British recommendations (7, 8).

A high variability in unfractionated heparin dose during hemodialysis has been previously reported (16). The cross-sectional study evaluating unfractionated heparin dosing at patient and facility level in 17,722 elderly American hemodialysis patients identified a significant increase in heparin dose for patients using a central venous catheter as their vascular access. In line with these results, loading and hourly infusion doses of UFH were also significantly higher in patients with a tunneled central venous catheter (versus arteriovenous access) in our cohort. The difference in total UFH dose per hemodialysis session according to vascular access was further emphasized by the longer infusion time in case of catheter use. The granularity of our dataset enabled to identify that catheter use rather than prior access dysfunction is associated with UFH dose escalation. Significant higher doses of heparin were also used in patients with well-functioning catheters and no history of access dysfunction.

A poor correlation between aPTT and ACT has been shown after unfractionated heparin use during cardiac catheterization (17, 18) or in a general ICU setting (19). In the current study, biological effects of UFH administration during chronic hemodialysis were monitored using aPTT and ACT ratios. According to ACT ratios, the majority of patients were adequately anticoagulated at 1 h and insufficiently anticoagulated at dialysis session end. Ratios of aPTT were consistently higher than ACT ratios, irrespective of dialysis access and whether coagulation tests were assessed 1 h after dialysis start or at treatment end. This resulted in the majority of patients fulfilling the definition of excessive anticoagulation (i.e., ratio > 2.5) according to aPTT ratio both at 1 and 4 h. The positive mean of the difference between aPTT an ACT correspond to a systematic bias in favor of higher aPTT ratios. Dose adjustments based on ACT will thus lead to supratherapeutic aPTT, known to be associated with bleeding risk. Of interest, a recent cross-sectional study identified a discordance between anti-Xa and aPTT assessing UFH anticoagulation and showed that adult patients more often had subtherapeutic aPTT with therapeutic anti-Xa levels (15). It is well recognized that aPTT and ACT have different sensitivities to heparin and moreover, that aPTT sensitivity to heparin is variable according to the reagent used. While aPTT testing might lack sensitivity when heparin doses are high, ACT may lack accuracy when heparin doses are low. Nevertheless, the overall high UFH doses used, the discrepant results in ACT and aPTT ratios, the low clotting scores and the high aPTT ratios in the majority of our patients suggest a true excess in anticoagulation during hemodialysis, although UFH doses appear correctly adjusted within our population using the ACT targets of adequate anticoagulation.

The identification of the ideal test in monitoring UFH administration to prevent clotting of the extracorporeal circuit is beyond the scope of this study. The lack of agreement between aPTT and ACT and the absence of a sound clinical indication of rapid coagulation test results in the setting of long-term and repeated unfractionated heparin administration during chronic hemodialysis, however, at least questions the usefulness of ACT testing during regular hemodialysis practice.

Overall, a quarter of sessions presented more than 20% loss in blood compartment volume after dialysis, a cut-off that has been associated with significant dialyzer clearance reduction in case of reuse of dialyzers (20). If clotting of the dialyzer’s capillaries is not a result of insufficient anticoagulation of the extracorporeal circuit, causes of impaired flow within the dialyzer need to be identified and screened for (e.g., Insufficient de-airing and priming of the extracorporeal circuit). Although we do not really know what caused the UFH dose increase in individual patients in our cohort, to reduce harms of the anticoagulant treatment during chronic hemodialysis, therapeutic targets of anticoagulation of the extracorporeal circuit should be clearly defined. Of interest, higher UFH doses during hemodialysis did not prevent blood compartment loss, in line with the results of a previous prospective observational trial (10). In this trial, a conversion from regular (6178 IU UFH/session) to low heparin dose (2913 IU UFH/session) in 66 prevalent hemodialysis patients resulted in similar small solute clearance, similar dialyzer clotting and dialyzer reuse rates and in a 25% dose reduction in erythropoietin stimulating agents (10). Our trial data did not inform on heparin exposure over time nor on biological anticoagulatory effect over time. This cross-sectional study design is inappropriate to assess associations between heparin dosing over time and bleeding events, EPO and iron consumption. Similarly, no data on prior hemodialysis sessions were included in the current analysis. Because the total unfractionated heparin dose administered during the studied hemodialysis session is a result of what happened during prior hemodialysis sessions, the current study cannot conclude on prior access flow rates and blood pump alarm events as predictive factors in heparin dosing.

We did not screen for antithrombin III deficiency, a state which would result in the impossibility for unfractionated heparin to exert anticoagulant effect hence prolong aPTT. Both the presence of lupus anticoagulant or seeding of an UFH-containing catheter lock would result in longer baseline aPTT and ACT results decreasing the aPTT or ACT ratio. All baseline aPTT and ACT results were taken into account in our analysis irrespective of whether baseline values were within or above normal reference range. The true effect of excessive anticoagulation might therefore even be underestimated with falsely lowered aPTT and ACT ratios in patients with baseline coagulation test values above normal range. Limitations in clinical and biological UFH monitoring protocols during chronic hemodialysis might increase the risk for insufficient or excessive anticoagulation. Our results emphasize the need to identify these shortcomings.

In conclusion, UFH dose during chronic hemodialysis in our cohort is highly variable and highest in patients with a catheter vascular access, even if the vascular access functions well. The high UFH doses, a lack of agreement between ACT and aPTT results and almost absent clotting complications support the hypothesis of true excessive anticoagulation during hemodialysis in our cohort. The risk for excessive anticoagulation is increased in case of ACT-based dose adjustments given the systematic bias in favor of higher aPTT ratios. Future research is needed to identify and contain the risks of repetitive and long-term anticoagulation during chronic hemodialysis and to explore optimal anticoagulation strategies for hemodialysis.

## Data availability statement

The datasets presented in this study can be found in online repositories. The names of the repository/repositories and accession number(s) can be found below: https://doi.org/10.5281/zenodo.5007102.

## Ethics statement

The studies involving human participants were reviewed and approved by the Ethics Committee of Universitair Ziekenhuis Brussel Laarbeeklaan 101, 1090 Brussels, Belgium Reference 2019/428. Written informed consent for participation was not required for this study in accordance with the national legislation and the institutional requirements.

## Author contributions

MD, KMW, and KF: study design and data analysis and interpretation. MD and KF: data collection and drafting the manuscript. DD, AT, M-LC, TR, and KMW: critical revision of the manuscript. All authors: final approval of the version to be published.
